# Chimeric antigen receptor T cells secreting anti-PD-L1 antibodies more effectively regress renal cell carcinoma in a humanized mouse model

**DOI:** 10.18632/oncotarget.9114

**Published:** 2016-04-29

**Authors:** Eloah Rabello Suarez, De-Kuan Chang, Jiusong Sun, Jianhua Sui, Gordon J. Freeman, Sabina Signoretti, Quan Zhu, Wayne A. Marasco

**Affiliations:** ^1^ Department of Cancer Immunology and Virology, Dana-Farber Cancer Institute (DFCI), Boston, MA, USA; ^2^ Department of Medicine, Harvard Medical School, Boston, MA, USA; ^3^ Department of Biochemistry, Faculdade de Medicina do ABC, Av. Príncipe de Gales, SP, Brazil; ^4^ National Institute of Biological Sciences, ZGC Life Science Park, Changping, Beijing, China; ^5^ Department of Medical Oncology, Dana-Farber Cancer Institute, Boston, MA, USA; ^6^ Department of Pathology, Brigham and Women's Hospital, Harvard Medical School, Boston, MA, USA

**Keywords:** immune checkpoint inhibitor, T cell exhaustion, chimeric antigen receptor, carbonic anhydrase IX, interleukin-21

## Abstract

Advances in the treatment of metastatic clear cell renal cell carcinoma (ccRCC) have led to improved progression-free survival of many patients; however the therapies are toxic, rarely achieve durable long-term complete responses and are not curative. Herein we used a single bicistronic lentiviral vector to develop a new combination immunotherapy that consists of human anti-carbonic anhydrase IX (CAIX)-targeted chimeric antigen receptor (CAR) T cells engineered to secrete human anti-programmed death ligand 1 (PD-L1) antibodies at the tumor site. The local antibody delivery led to marked immune checkpoint blockade. Tumor growth diminished 5 times and tumor weight reduced 50–80% when compared with the anti-CAIX CAR T cells alone in a humanized mice model of ccRCC. The expression of PD-L1 and Ki67 in the tumors decreased and an increase in granzyme B levels was found in CAR T cells. The anti-PD-L1 IgG1 isotype, which is capable of mediating ADCC, was also able to recruit human NK cells to the tumor site *in vivo*. These armed second-generation CAR T cells empowered to secrete human anti-PD-L1 antibodies in the ccRCC milieu to combat T cell exhaustion is an innovation in this field that should provide renewed potential for CAR T cell immunotherapy of solid tumors where limited efficacy is currently seen.

## INTRODUCTION

One of the new emerging mechanisms associated with the progression of clear cell renal cell carcinoma (ccRCC) and other tumors is the immune checkpoint pathway, which consists of cellular interactions that prevent excessive activation of T cells under normal conditions. As an evasion mechanism, many tumors are able to stimulate the expression of immune checkpoint molecules, resulting in an exhausted phenotype of T cells that cannot restrain tumor progression [1]. Emerging clinical data highlight the importance of one inhibitory ligand and receptor pair as an immune checkpoint: the programmed death-ligand 1 (PD-L1; B7-H1 and CD274) and programmed death receptor-1 (PD-1; CD279), in preventing the killing of cancer cells by cytotoxic T-lymphocytes. The PD-1 receptor is expressed by activated T cells, B cells and myeloid cells [2], whereas PD-L1 is expressed on antigen-presenting cells including human peripheral blood monocytes stimulated with interferon-γ (IFNγ) as well as on activated B cells and dendritic cells. In addition, PD-L1 is expressed constitutively on non-lymphoid tissues such as the heart, placenta, skeletal muscle and lung where it may serve to downregulate T cell receptor (TCR) signaling in PD-1+ cytotoxic T-lymphocytes to safeguard against autoimmune mediated tissue damage [2]. PD-L1 overexpression is also found on many tumor types [3] and mediates an immunosuppressive function through its interaction with PD-1 and other proteins, including CD80 (B7.1). Immunomodulatory human/humanized mAbs that target the PD-1/ PD-L1 axis have shown durable tumor control in clinical trials [4, 5] and good safety profiles [6]. An Fc-engineered anti-PD-L1 mAb that lacks FcγR engagement has shown tumor regression in RCC, with a 12% of objective response rate, 41% stable disease lasting at least 24 weeks and 53% of progression-free survival at 24 weeks [4]. Furthermore, the immune checkpoint inhibitors have the advantage of facilitating a memory response unlike the existing cytotoxic and targeted cancer therapies [7].

A modified TCR called chimeric antigen receptor (CAR) containing single chain variable antibody fragment (scFv) previously selected for high affinity against a tumor-associated antigen is a powerful new approach against cancer. The scFv presented in the first-generation CAR is linked to the intracellular signaling motif of CD3ζ that facilitates T cell activation following antigen binding. In second-generation CARs, the scFv is linked to the signaling co-stimulatory endodomains of CD28, 4-1BB, or OX40 to CD3ζ, and third-generation CARs have two of these elements linked to CD3ζ in tandem [8]. These co-stimulatory endodomains provide complete T cell activation during TCR recognition by antigen-presenting cells (APCs), improving cytokine production and proliferation of CAR T cells [9]. A prominent example of a clinically successful CAR T cell therapy for the management of B cell malignancies is the second-generation CD19-specific CAR encoding CD28 or 4-1BB signaling moieties, which has shown antitumor activity in clinical trials [10–12]. However, the effect of CAR T cells has been modest for the treatment of solid tumors due to several factors including the difficulty in identifying unique tumor associated antigens, inefficient homing of CAR T cells to tumor locations, their low persistence after infusion and their functional impairment in the immunosuppressive microenvironment of the solid tumors [13].

In an effort to counteract the negative effects of the tumor microenvironment on CAR T cell function, we designed and tested a self-inactivating (SIN) bicistronic lentiviral vector that could deliver a new type of double immunotherapy based on enabling targeted anti-carbonic anhydrase (CAIX) CAR T cells to secrete anti-PD-L1 antibodies at the tumor site to block T cell exhaustion. CAIX was chosen as a target to our CAR because this enzyme is overexpressed in many hypoxic solid tumors [14, 15], being the most well-characterized tumor-associated antigen overexpressed in ccRCC [16, 17]. Our results support the feasibility of this approach for the improved treatment of ccRCC which has the potential to be extended to other solid tumors.

## RESULTS

### Characterization of anti-CAIX CAR T cells secreting anti-PD-L1 IgG1 or IgG4

**Figure 1:**
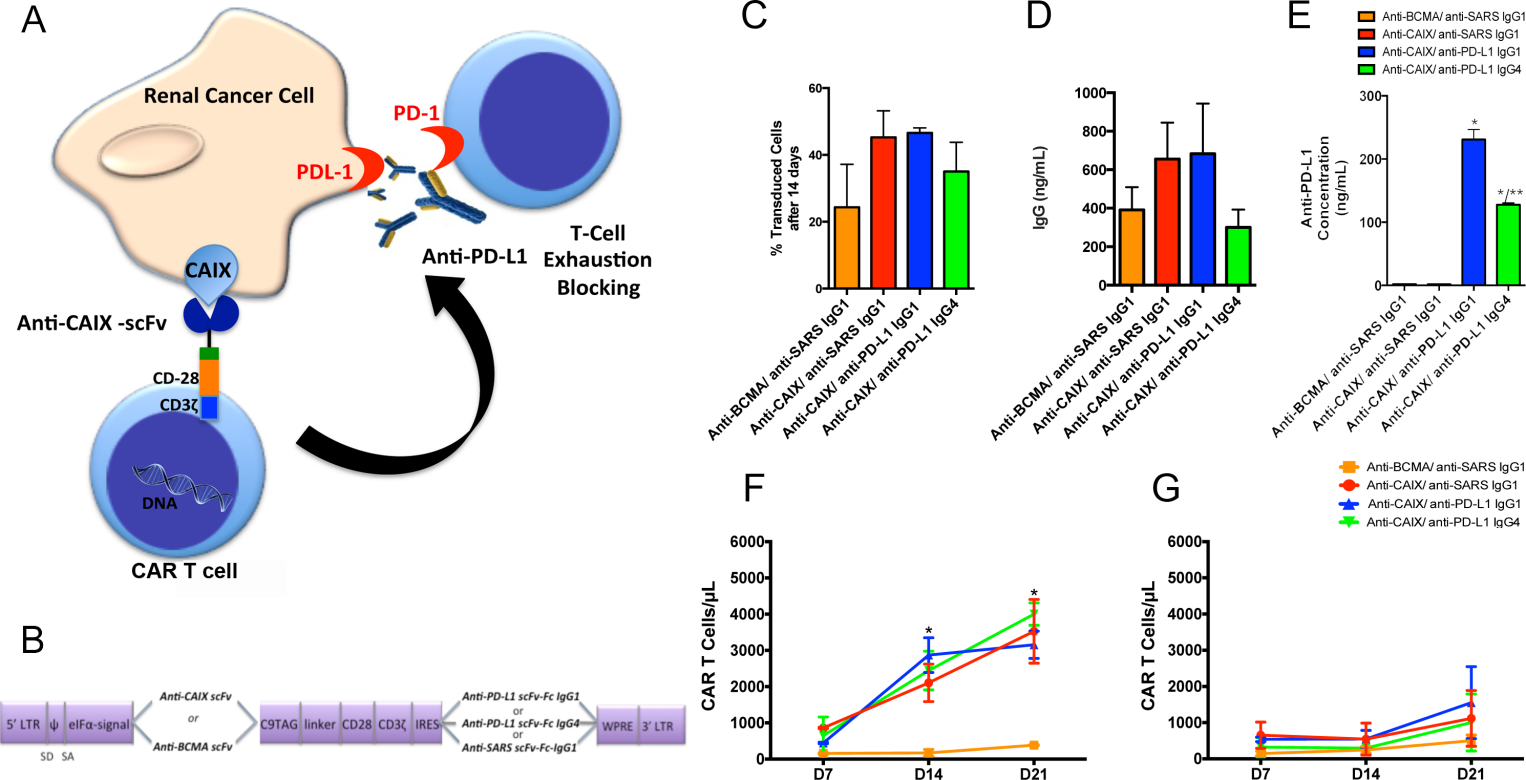
Chimeric antigen receptors (CAR) constructs for CD8+ T cells transduction (**A**) T cells were transduced with the lentiviruses to generate anti-CAIX CAR T cells, which are able to recognize CAIX positive RCC, and also secrete anti-PD-L1 IgG1 or IgG4 in the tumor microenvironment to block PD-1/PD-L1-induced T cell exhaustion. (**B**) Schematic representation of pHAGE lentiviral vectors encoding second-generation CARs fused with CD28 co-stimulatory endodomain. The anti-carbonic anhydrase IX (CAIX) or the Anti-B-cell maturation antigen (BCMA) scFv (as a negative control) were inserted after the eIFa promoter in order to express the CAR binding domain. The second cassette, after the Internal Ribosome Entry Site (IRES) sequence, encodes the secretable anti-PD-L1 IgG1 or IgG4 isotypes or the anti-severe acute respiratory syndrome (SARS) coronavirus IgG1 (negative control). LTR: long terminal repeat, eIFα: eukaryotic initiation factor alpha, scFv: single-chain variable fragment, C9 TAG: C9 peptide TETSQVAPA, IRES: Internal Ribosome Entry Site, WPRE: Woodchuck Hepatitis Virus Posttranscriptional Regulatory Element. (**C**) Percentage of CAR T cells 14 days after transduction, representing the stable long-term expression of CAR by the integrated lentiviruses in CD8+ T cells. The CD8+ T cells were selected using Dynabeads CD8 Positive Isolation Kit (Life Technologies) and activated with Dynabeads Human T Cell Activator CD3/CD28 (Life Technologies) in the presence of IL-21 50 U/mL. IL-21 was added to the medium every 2 days. After 14 days, the CAR T cells were incubated with human CAIX-Fc or BCMA-Fc, followed by incubation with an APC conjugated anti-human Fc IgG (Southern Biotech) or goat-anti-mouse IgG Ab (Biolegend) and analyzed by flow cytometry. (**D**) Concentration of IgG secreted into the medium of transduced T cells evaluated by Human IgG ELISA Quantitation Set (Bethyl Laboratories). (**E**) Concentration of anti-PD-L1 antibodies in the supernatant of 293T Cells transduced with lentiviruses containing anti-CAIX or anti-BCMA CAR and anti-PD-L1 IgG1, anti-PD-L1 IgG4 or irrelevant anti-SARS IgG1 sequences. The antibodies in the supernatant were purified, biotinylated and incubated with 5 μg/mL of human PD-L1 pre-immobilized in the 96 wells MaxiSorp plate (Nunc). The biotinylated antibodies were detected by incubation with streptavidin-HRP and developed with TMB. The absorbance was read at λ = 450 nm. *P < 0.001 compared to anti-BCMA CAR/anti-SARS IgG1 and anti-CAIX CAR/anti-SARS IgG1. **P < 0.05 compared to anti-CAIX CAR/anti-PD-L1 IgG1 (**F** and **G**) Clonal expansion of CD8+ CAR T cells. (**F**) CAR T cells in the presence of skrc52 CAIX+/PD-L1- cells over time. *P < 0.05 comparing all CARS to anti-BCMA CAR/anti-SARS IgG1. (**G**) CAR T cells in the presence of skrc52 CAIX-/PD-L1- cells over time. After activation with Dynabeads Human T Activator CD3/CD28 (Life Technologies) for five days, the CAR T cells were cultured with skrc52 CAIX+/PD-L1- or skrc52 CAIX-/PD-L1- and IL-21 (50 U/mL), which was added to the medium every 2 days for 21 days. The results represent the average ± SD of three donors in duplicate.

In order to develop a new CAR therapy for CAIX+ RCC that could block T cell exhaustion (Figure 1A), we engineered a bicistronic lentiviral vector to express the anti-CAIX (G36) scFv linked to CD28 and CD3-ζ signaling domains (G36-CD28z CAR) in the first cassette and anti-PD-L1 IgG1 or IgG4 in a second expression cassette after an IRES site (Figure 1B). As controls, we used an anti-CAIX CAR or anti-B cell maturation antigen (BCMA) CAR construct secreting an irrelevant anti-severe acute respiratory syndrome (SARS) IgG1 mAb. The lentiviruses generated from these constructs were transduced into CD8 T cells and cultivated in the presence of IL-21, which yielded CAR T cells with modestly improved proliferation than was seen with IL-2 (Supplementary Figure 1A and 1B), while maintaining the same specific killing activity for CAIX+ RCC (Supplementary Figure 1C and 1D). The superiority of IL-21 compared with IL-2 to induce T cells proliferation was previously described [18, 19]. The percentage of CAIX and PD-L1 expression in all RCC lineages used in our experiments is shown in Supplementary Figure 2.

The CAR T cell functionality was demonstrated in Supplementary Figure 3, where CD8 T cells transduced with all CARs were able to proliferate in the presence of IL-21 and anti-CD8/CD28 beads (Supplementary Figure 3A and 3B), achieving transduction levels of 65–90% after four days (Supplementary Figure 3C). Fourteen days after transduction, we evaluated the stable long-term expression of CAR by the integrated lentiviruses (Figure 1C), which was maintained around 25–50% for all CARs. Total IgG levels secreted by transduced CD8+ T cells was also determined, ranging around 300–650 ng/mL after 4 days (Figure 1D). The binding specificity of the anti-PD-L1 IgG1 and IgG4 antibodies for human PD-L1 was also confirmed (Figure 1E). The ability of anti-CAIX CAR T cells to undergo clonal expansion exclusively in the presence of CAIX+ RCC cells was established (Figure 1F). Anti-CAIX CAR T cells cannot expand significantly in the presence of CAIX- RCC cells (Figure 1G).

### Effector activity of Anti-CAIX CAR T cells

**Figure 2:**
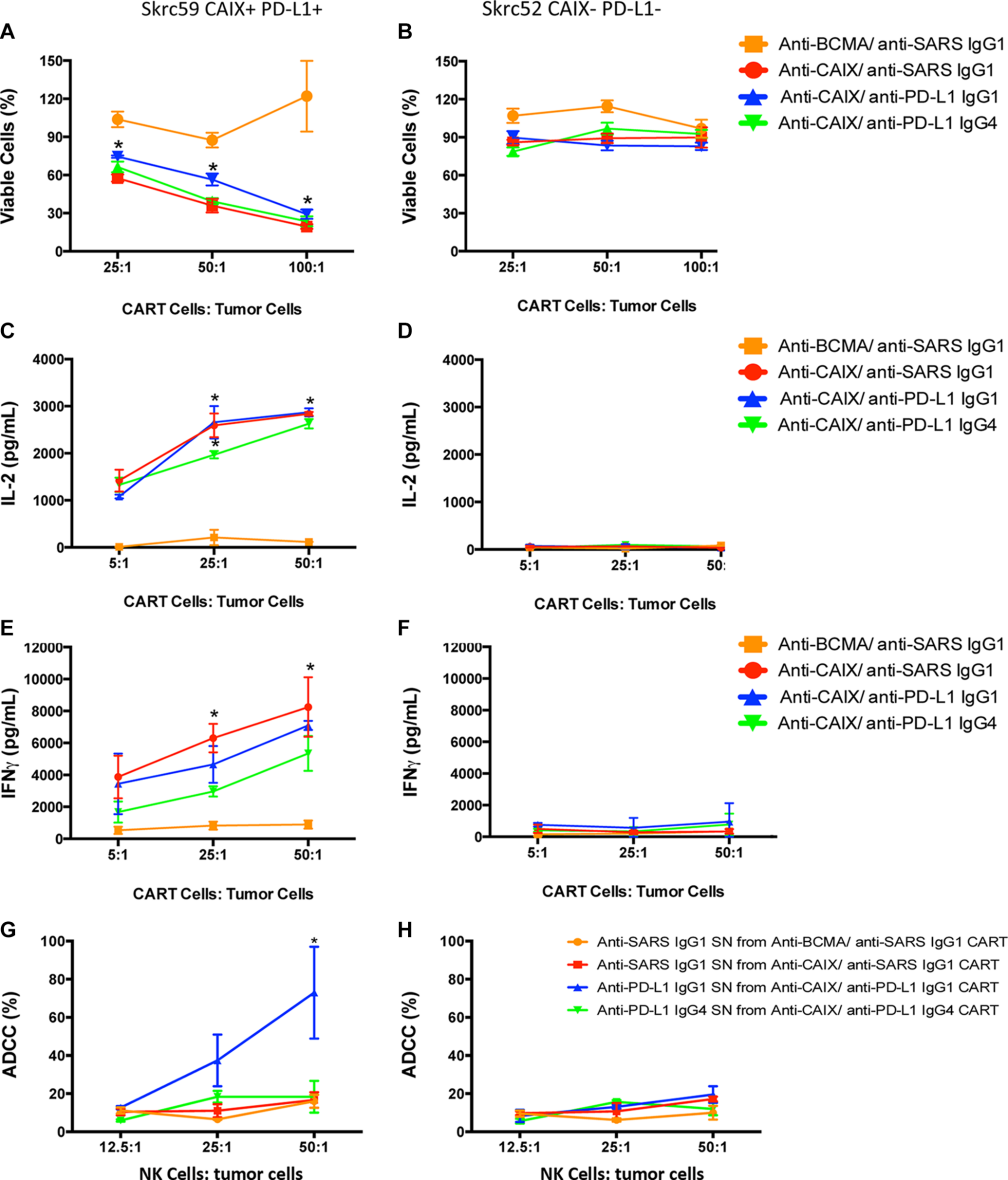
CAR T cell effector function (**A**) Viability of skrc59 CAIX+/PD-L1+ cells or (**B**) Skrc52 CAIX-/PD-L1- cells incubated ON with each indicated CAR T cells. These CAR T cells were used 4 days after lentiviral transduction. The cell viability was evaluated by MTT (Molecular Probes). *P < 0.05 for all CAR T cells compared to anti-BCMA CAR/anti-SARS IgG1. (**C**) IL2 released by the CAR T cells after overnight contact with skrc59 CAIX+/PD-L1+ cells or (**D**) Skrc52 CAIX-/PD-L1- cells. The IL-2 secretion was evaluated using the Human IL-2 ELISA Ready-SET-Go Kit (eBioscience). *P < 0.001 for all CAR T cells compared to anti-BCMA CAR/anti-SARS IgG1. (**E**) IFNγ released by the CAR T cells after overnight contact with skrc59 CAIX+/PD-L1+ or (**F**) Skrc52 CAIX-/PD-L1- cells. The IFNγ secretion was evaluated using the Human IFNγ ELISA Ready-SET-Go Kit (eBioscience). *P < 0.05 for all CAR T cells compared to anti-BCMA CAR/anti-SARS IgG1. (**G**) Ab-dependent cell-mediated cytotoxicity (ADCC) of skrc59 CAIX+/PD-L1+ cells or (**H**) skrc52 CAIX-/PD-L1- cells after incubation with the supernatant (SN) of the CAR T cells containing 500 ng/mL of the anti-PD-L1 IgG1, anti-PD-L1 IgG4 or the anti-SARS IgG1. NK cells were purified using an EasySep^™^ Human NK Cell Enrichment Kit (StemCell^™^ Technologies) from PBMC. RCC cell lines skrc59 CAIX+/PD-L1+ and skrc52 CAIX-/PD-L1- were used as target cells and plated at 1.5 × 10^3^/well in a 96-well plate. RCC cells were incubated with 50 μL of the CAR T cells supernatant adjusted for 500 ng/mL of the respective Ab by 1 hour. After the incubation, the cells were washed with medium and incubated with 12.5:1, 25:1 or 50:1 NK cells:tumor cells for 4 h, 37°C. Culture supernatants were harvested by centrifugation and lactate dehydrogenase (LDH) measured in the supernatant by CytoTox 96^®^ Non-Radioactive Cytotoxicity Assay (Promega) at 490 nm. These results represent the average ± SD of three donors in duplicate.

All anti-CAIX CAR T cells were able to induce around 50–70% decrease in the viability of Skrc59 CAIX+/PD-L1+ cells, indicating that the anti-PD-L1 IgG1 and IgG4, secreted by CAR T cells, did not augment cell killing under these assay conditions (Figure 2A). The effect of anti-CAIX CAR without anti-PD-L1 secretion was also analyzed previously in another natural CAIX+ ccRCC cell line skrc52; however this cell type was not studied herein due to its lack of PD-L1 expression [20]. The anti-CAIX CAR T cells only produced IL-2 and IFNγ in the presence of CAIX+/PD-L1+ cells, demonstrating the CAIX dependent activation of these CAR T cells (Figure 2C and 2E, respectively). For the anti-CAIX CAR T cells secreting anti-PD-L1 IgGs, a unique differential effect was seen for the IgG1 isoform, which was able to induce around 60% of ADCC in CAIX+/PD-L1+ RCC cells when incubated with natural killer cells (NK) (Figure 2G). No effect on cell viability, cytokine secretion or ADCC was detected for any of the CAR T cells in the presence of CAIX-/PD-L1- cells (Figure 2B, 2D, 2F and 2H).

### Anti-CAIX CAR T cells secreting anti-PD-L1 antibodies can diminish T cell exhaustion *in vitro*

**Figure 3:**
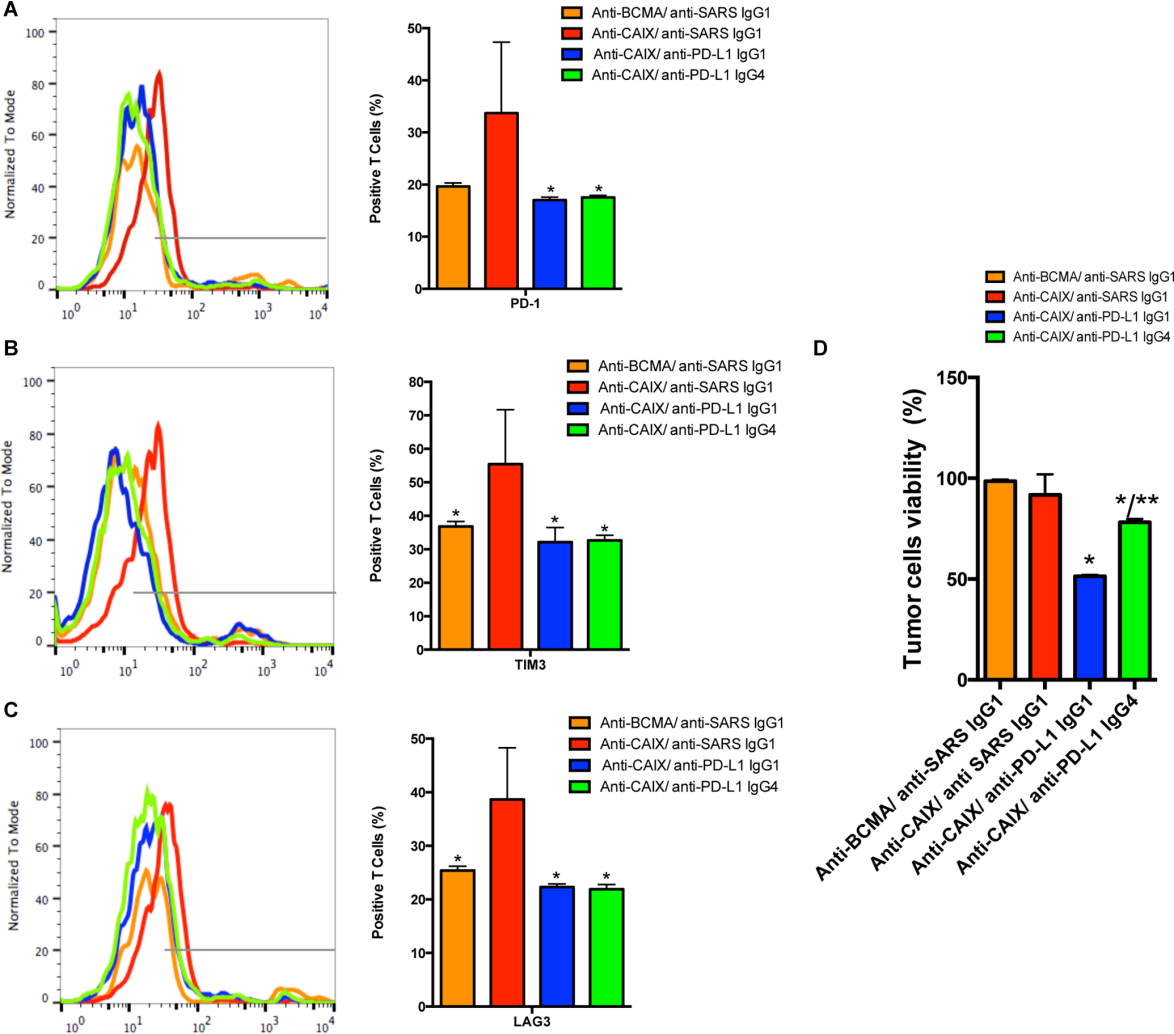
The CAR T cell expression of exhaustion markers PD-1 (**A**), TIM-3 (**B**) or LAG-3 (**C**). *P < 0.05 compared to Anti-CAIX CAR/anti-SARS IgG1. The CAR T cells were cultured in the presence of IL-21 50 U/mL and Dynabeads Human T Activator CD3/CD28 for 5 days. After this period the CAR T cells were co-cultured with skrc59 CAIX+/PD-L1+ for 9 days. In order to evaluate the status of the CAR T cell exhaustion, the CAR T cells were stained with FITC-conjugated anti-human PD-1, PE-conjugated anti-human TIM-3 and PerCP/Cy5.5 anti-human LAG-3 and analyzed by flow cytometry. (**D**) Viability of skrc59 CAIX+/PD-L1+ cells after incubation with exhausted CAR T cells ON (50:1 E:T). The cell viability was evaluated by MTT (Molecular Probes). The number of CAR T cells was correct by the % of transduction for this experiment. *P < 0.05 compared to both anti-CAIX CAR/anti-SARS IgG1 and anti-BCMA CAR/anti-SARS IgG1. **P < 0.05 compared to anti-CAIX CAR/anti-PD-L1 IgG1. These results represent the average ± SD of three donors.

An approximate 50% decrease in the exhaustion markers LAG-3, TIM-3 and PD-1 was found in the anti-PD-L1 IgG1- and IgG4-secreting anti-CAIX CAR T groups (Figure 3A–3C, respectively) after induction of exhaustion compared to the parental anti-CAIX. At this point, the killing activity of the anti-CAIX CAR T cells was retested against skrc59 CAIX+/PD-L1+ cells. As shown in Figure 3D, the anti-CAIX CAR T cells without anti-PD-L1 had lost their killing activity against CAIX+/PD-L1+ RCC *in vitro* to a level that was similar to the irrelevant CAR group, establishing that these anti-CAIX CAR T cells had become exhausted. In contrast, the RCC viability was reduced to 50% in the anti-CAIX CAR T/anti-PD-L1 IgG1 group and 25% in the anti-PD-L1 IgG4 CAR T group, respectively. These data provide evidence that the checkpoint blockade elicited by the secreted anti-PD-L1 IgGs can lead to diminished T cell exhaustion.

### Anti-CAIX CAR T cells secreting anti-PD-L1 antibodies can further decrease tumor growth in an orthotopic mouse model of human RCC

**Figure 4:**
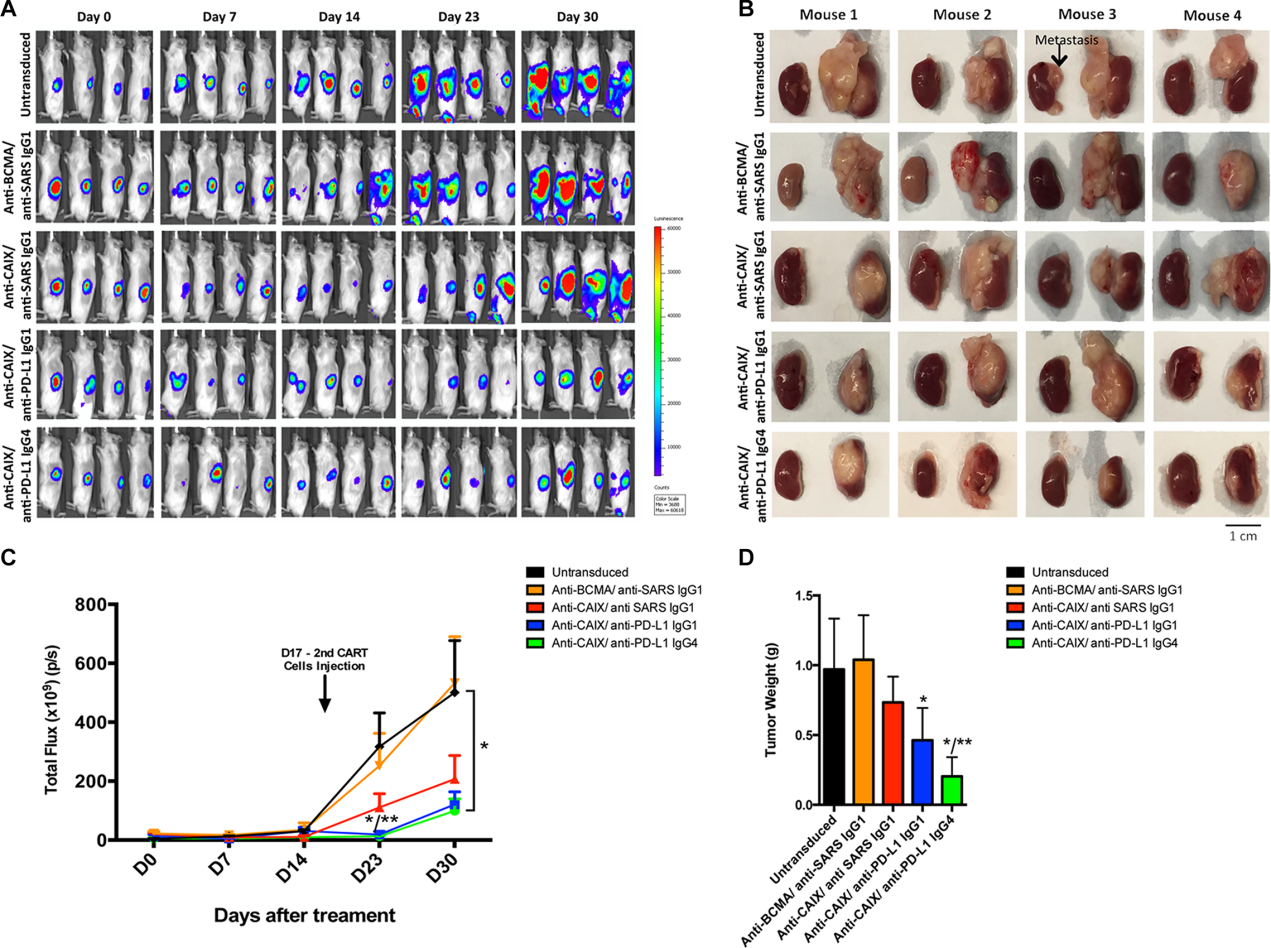
Effects of the CAR T cells in an orthotopic model of human ccRCC (**A**) NSG Mice (*N* = 30) were injected with 5.0 × 10^4^ skrc59 CAIX+/PD-L1+/luciferase+RCC cells. After a week, the mice were injected by i.v. with 1.0 × 10^7^ CAR T or untransduced T cells (Day 0). The CAR T cells were generated by transduction with the lentiviral vectors encoding: anti-CAIX CAR/anti-PD-L1 IgG1, anti-CAIX CAR/anti-PD-L1 IgG4, anti-CAIX CAR/anti-SARS IgG1, or anti-BCMA CAR/anti-SARS IgG1 (*N* = 6 mice per group). Tumor growth was quantified by bioluminescence imaging after 5 minutes of luciferin IP injection using IVIS on Day 0 before, and on Days 7, 14, 23 and 30 after the first CAR T cells injection. A second injection of 2.5 × 10^6^ cells was made on Day 17. (**B**) Imaging of the tumors after excision on Day 30 with RCC-implanted kidney on the right side of each image. Scale bar = 1 cm. (**C**) Tumor growth curve. *P < 0.05 when anti-PD-L1 IgG1 and IgG4 groups were compared to anti-BCMA CAR/anti-SARS IgG1 and **P < 0.05 when anti-PD-L1 IgG1 and IgG4 groups were compared to anti-CAIX CAR/anti-SARS IgG1. (**D**) Average tumor weight after 30 days of treatment. *P < 0.05 compared with anti-BCMA CAR/anti-SARS IgG1 CAR, **P < 0.05 compared with anti-CAIX CAR/anti-SARS IgG1. Animal experiments were performed in accordance with the guidelines of the DFCI Animal Care Committee.

NSG mice were used to establish an orthotopic RCC model by injecting skrc59 CAIX+/PD-L1+/luciferase+ RCC cells under the kidney capsule followed by an i.v. injection of 1.0 × 10^7^ CAR T or untransduced T cells (Day 0) and repeated treatment on Day 17 with a lower dose (2.5 × 10^6^) of the same cells. We did not treat the mice with systemic IL-2 to maintain the proliferation of CAR T cells to avoid the bias that this molecule could exert on the tumor growth. The data in Figure 4A–4C demonstrate that all three anti-CAIX CAR T cell groups showed decreased RCC growth compared to irrelevant anti-BCMA CAR T cells or untransduced cells over the course of the experiment. The marked anti-tumor effects exhibited by the anti-CAIX CAR T cells secreting anti-PD-L1 IgG1 or IgG4 become evident on Days 23 and 30 (Figure 4A and 4B). However, even one week after i.v. treatment with CAR T cells, we observed that the tumors were 2-3 times smaller in the anti-PD-L1-secreting CAR T cells when compared with parental anti-CAIX CAR T cells and the two control groups (Supplementary Figure 4A). We also analyzed CD45+ T cell survival in the mouse blood to gauge their survival in this passive transfer model. On Day 8 we observed that the proportion of human T cells within the PBMCs were only 10–15% (Supplementary Figure 4B). One week after the second injection (Day 23) the anti-PD-L1 IgG1 and IgG4 groups had tumors 15 times smaller than the control groups and 5 times smaller than the anti-CAIX CAR T cells without anti-PD-L1 secretion (Figure 4C and Supplementary Figure S4A). On Day 30, the group of mice treated with CAR T cells secreting anti-PD-L1 antibodies had tumors 5 times smaller than the control groups (Figure 4C and Supplementary Figure S4A). The excised tumor weights were also lower in the mice treated with CAR T cells secreting anti-PD-L1 antibodies, and this was particularly evident for the anti-PD-L1 IgG4 antibody group (Figure 4B and 4D).

### Analysis of CAR T cell tumor infiltration and evidence that anti-CAIX CAR T cells secreting anti-PD-L1 antibodies can lead to reversal of T cell exhaustion

**Figure 5:**
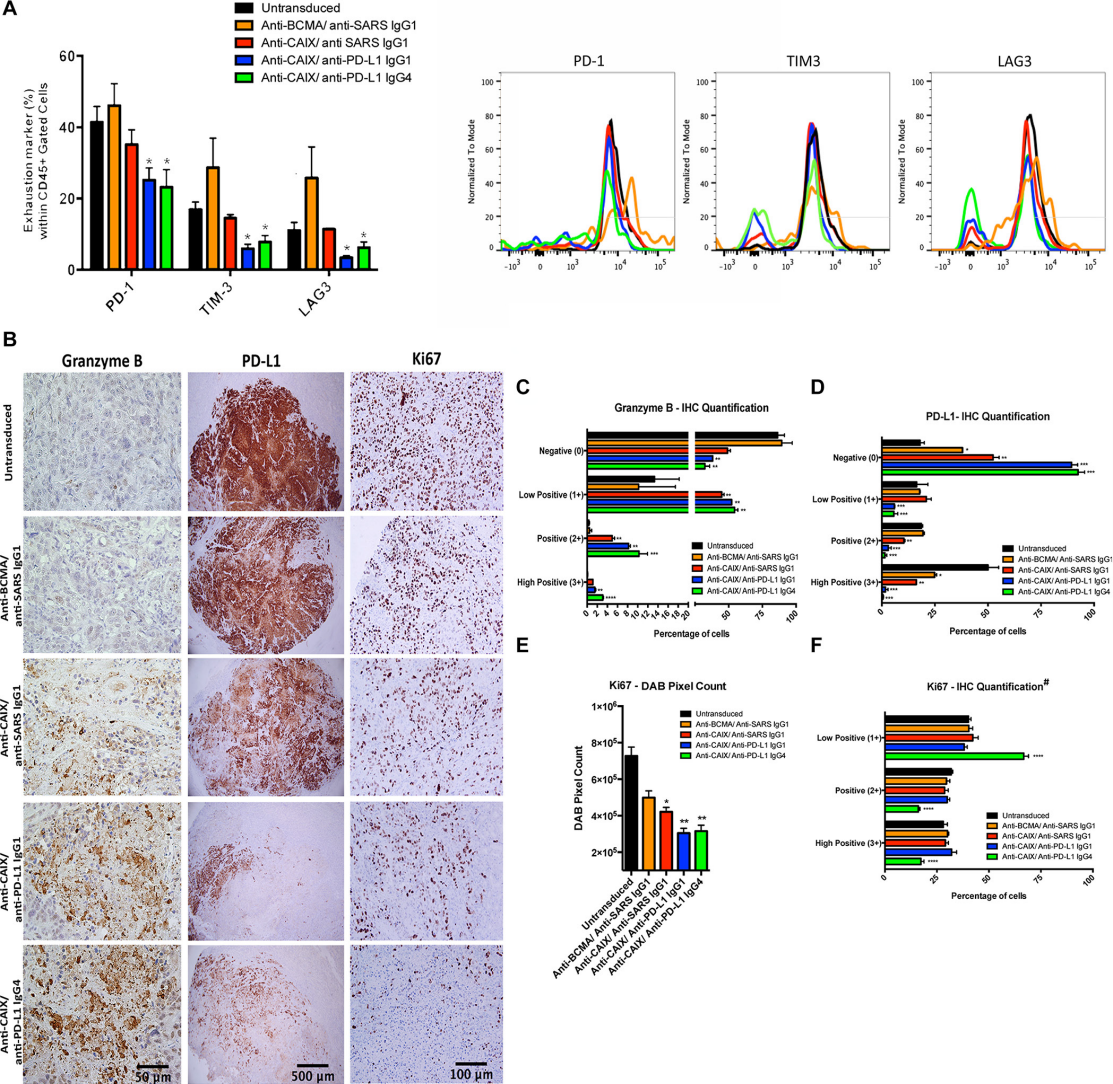
Exhaustion markers on tumor infiltrating lymphocytes (TIL) after CAR T cell treatment *in vivo* and immunohistochemical (IHC) analysis of CAR T cells antitumor activity (**A**) Expression of exhaustion markers in TILs. The kidney tumors from all mice were divided in two parts and one-half was fragmented in small pieces and digested with collagenase and DNAse for extraction of TIL. The single CAR T cell suspensions were analyzed for the exhaustion markers PD-1, TIM-3 and LAG-3. *P < 0.05 compared with untransduced, anti-BCMA CAR/anti-SARS IgG1 CAR and anti-CAIX CAR/anti-SARS IgG1. (**B**) IHC analysis of tumor sections by detection of granzyme B, PD-L1 and Ki67 expression. The fixed tumors were paraffin-embedded, sectioned at four-micrometer, dewaxed and rehydrated in a decreasing ethanol series. Endogenous peroxidase activity was quenched using 3% hydrogen peroxide. The antigen retrieval was performed using pressure cooker in citrate buffer (pH = 6.0) for 45 seconds at 123°C, 15 PSI. The tissues were stained with the anti human: Ki67 (Vector, VP-K451), PD-L1 (Clone 405.9A11, produced in Dr. Gordon Freeman's lab), granzyme B (Abcam, ab4059) or NCAM (CD56) (Abcam, ab133345) antibodies followed by secondary HRP conjugated anti-rabbit Ab or HRP-Avidin. The slides were developed using DAB and counterstained with hematoxylin. The images were obtained in an Olympus BX51 microscopy using a DP71 digital camera (Olympus) and analyzed in the DP Controller Software (Olympus). The scale bars represent the magnification of the images of each column [500 μm (40×), 100 μm (200×) or 50 μm (400×)]. (**C–F**) IHC Quantification. The images quantification was performed using the IHC Profiler Plugin of ImageJ Software [47]. The percentage of negative (0), low positive (1+), positive (2+) or high positive (3+) cells were shown. For granzyme B (**C**) and PD-L1 (**D**) quantification, cytoplasmatic quantification was applied where all the image DAB pixels were counted. For quantification of nuclear protein Ki67 (**F**), DAB staining pattern is confined to the nuclei and the threshold feature of ImageJ is used to select the positive-stained areas for quantification and non-staining nuclei are not recorded [47]. DAB total pixels were also shown (**E**) for evaluation of total Ki67 staining in all fields, including negative areas. *P < 0.05 compared with untransduced, **P < 0.05 compared with untransduced and anti-BCMA/anti-SARS IgG1, ***P < 0.05 compared with untransduced, anti-BCMA/anti-SARS IgG1 and anti-CAIX/anti-SARS IgG1 and ****P < 0.05 anti-CAIX/anti-PD-L1 IgG4 compared with all other groups.

Analysis of the excised tumors showed around 10% of tumor-infiltrating lymphocytes (TIL) in all groups (Supplementary Figure S4C). One of the most important effects observed with the anti-CAIX CAR T cells secreting anti-PD-L1 IgG1 or IgG4 antibodies *in vivo* was their ability to reduce the expression of the exhaustion markers PD-1, TIM-3 and LAG-3. As shown in Figure 5A, for the anti-PD-L1-secreting CAR T cells we observed a decrease of approximately 30%, 40–50% and 50–70% expression of PD-1, TIM-3 and LAG-3, respectively, compared to the parental anti-CAIX CAR T cell treated group. These data provide evidence that the locally secreted antibodies decreased the expression of surface markers that are associated with T cell exhaustion.

The effector activity of CAR T/TIL cells and their influence over RCC proliferation *in vivo* were also evaluated by immunohistochemical staining of the excised tumor tissue. Granzyme B staining showed the highest percentage of 2+/3+ positive cells in the RCC tumors treated with the anti-CAIX CAR T cells secreting anti-PD-L1 IgG4 followed by anti-PD-L1 IgG1-secreting cells, both of which were higher than parental anti-CAIX CAR T cells and other control groups (Figure 5B and 5C). In addition, PD-L1 expression decreased dramatically in the tumors treated with anti-CAIX CAR T cells secreting either anti-PD-L1 IgG isotype compared to parental anti-CAIX CAR T cells (Figure 5D). The anti-PD-L1 mAb used for IHC [21] recognizes a different domain than the anti-PD-L1 mAb secreted by the lentivirus, and the decrease in PD-L1 expression observed in the groups treated with anti-PD-L1-secreting CAR T cells is probably due to endocytosis or tumor cell death. Moreover, a greater decrease in Ki67 expression was observed in the anti-CAIX/anti-PD-L1 IgG-secreting groups compared to parental anti-CAIX CAR T cells as can be seen both in the IHC images (Figure 5B) and total DAB pixel count graph (Figure 5E). When the intensity of Ki67 expression was compared among the positive stained nuclei (Figure 5F), only the anti-CAIX/anti-PD-L1 IgG4 CAR T cell group showed a shift toward a greater number of low positive cells, suggesting that the anti-PD-L1 IgG4 isoform is more potent *in vivo* (green bars). These studies demonstrate that the anti-PD-L1-secreting CAR T cells were more effective at ccRCC killing *in vivo* and that this is, at least in part, explained by their decreased exhaustion.

### Anti-CAIX CAR T cells secreting anti-PD-L1 IgG1 antibodies can recruit NK cells to the tumor

**Figure 6:**
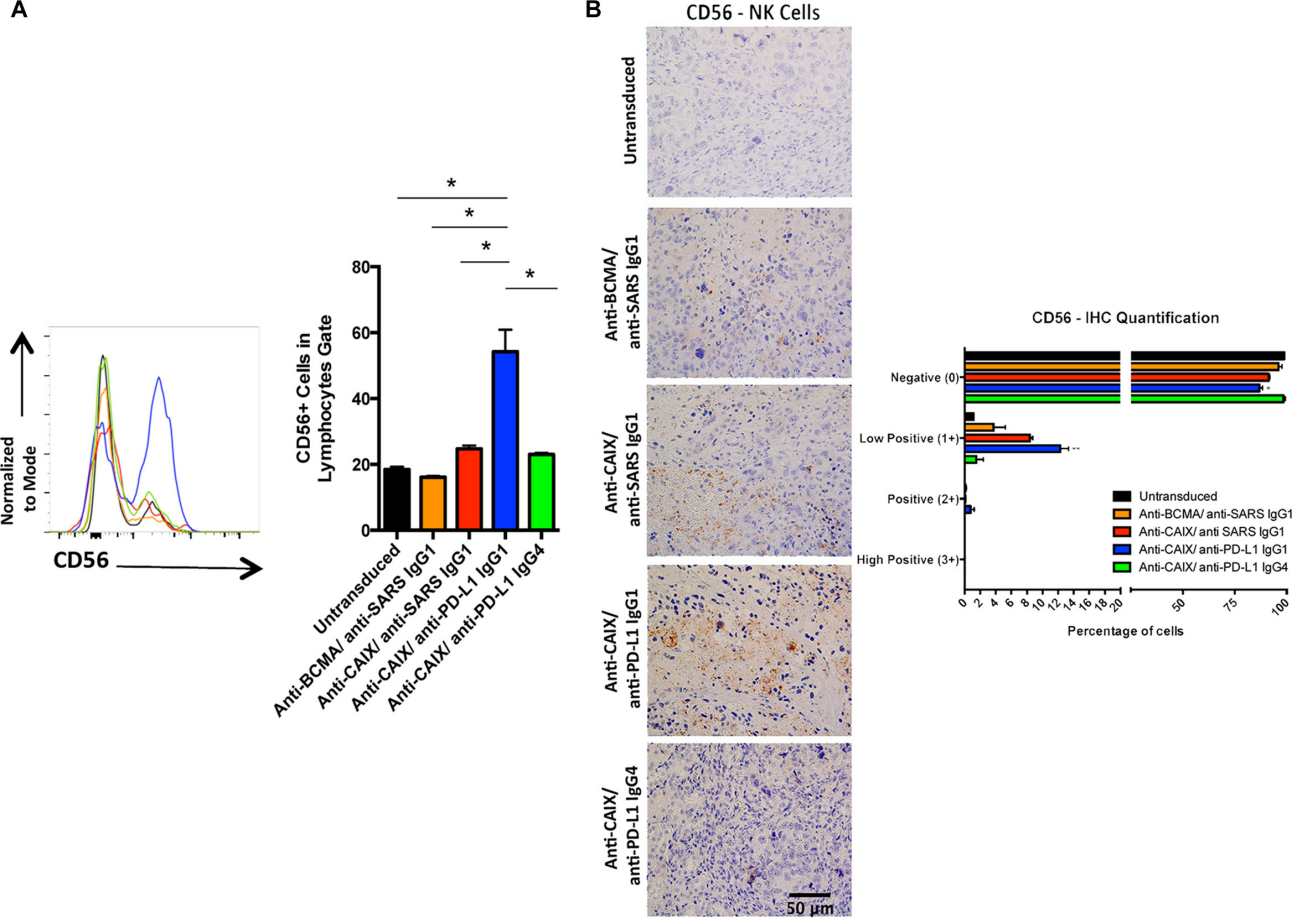
Human natural killer (NK) cells in the tumors treated with anti-CAIX CAR T cells secreting anti-PD-L1 IgG1 Ab (**A**) Percentage of CD56+ cells (NK marker) in the tumors. Two mice of each group were injected with 4.5 × 10^6^ NK cells from two different donors one day before the euthanasia. The kidney tumors from all mice were divided in two parts and one-half was fragmented in small pieces and digested with collagenase and DNAse to extract NK. NK cells present in the tumor were stained with APC-Anti-CD56 Ab and analyzed by flow cytometry (left panel). Statistical analysis showed that only the anti-CAIX/anti-PD-L1 IgG1 group had greater numbers of NK cell in the TILs, *P < 0.05 (right panel). (**B**) CD56+ cells in the excised tumors as detected by IHC (left panel) and quantified using IHC Profiler Plugin of ImageJ Software (right panel). The IHC was performed as described in Methods and the slides were stained with rabbit anti-human CD56 primary mAb 1:100 (Abcam, ab133345). The images were obtained in an Olympus BX51 microscopy using a DP71 digital camera (Olympus) and analyzed in the DP Controller Software (Olympus). The quantification was performed using the IHC Profiler Plugin of ImageJ Software [47]. The scale bars represent the magnification of the images (400×). *P < 0.05 compared with untransduced, **P < 0.05 compared with untransduced and anti-BCMA/anti-SARS IgG1.

To document that the secreted anti-PD-L1 antibodies were actively binding to the CAIX+/PD-L1+ tumor cells, we injected the mice with freshly isolated CD56+ NK cells 2 days before the euthanasia and then stained for NK cell accumulation in the tumor. As shown in Figure 6, staining of single cell suspensions of the TILs showed a marked and statistically significant presence of > 40% more NK cells in the tumors of mice that were treated with anti-CAIX/anti-PD-L1 IgG1 CAR T cells when compared with the other group including anti-CAIX CAR T cells that secrete anti-PD-L1 IgG4 cells that does not bind to FcγRIIIa on human NK cells. The increase in NK cells in the anti-CAIX/anti-PD-L1 IgG1 group TILs was also detected and quantified by IHC (Figure 6). These data provide strong evidence that the secreted anti-PD-L1 IgG1 antibody maintained local PD-L1 target recognition and was able to recruit NK effect cells to the tumor site.

## DISCUSSION

In this study, we focused our efforts on developing a single targeted CAR T cell immunotherapy that had the capacity to change the tumor microenvironment, which is known to contribute to immune escape of solid tumors. We built our immunotherapy platform based on the scientific principles that are showing clinical success [6, 22]. This is important since CAR T cells are not immune to dysregulation once they reach the cancer site due to tumor cell contact and the complex soluble mediator milieu that can impact their function. Indeed, changing this microenvironment is the basis of encouraging clinical responses with mAb therapies that are aimed at restoring T cell anti-tumor immunity [22]. Engineering CAR T cells to secrete and deliver high concentrations of human mAbs against molecules involved in immune checkpoint blockade at the tumor site could diminish their potential to become exhausted as well as lead to *trans* reversal of exhaustion for the CD8+TILs and other immune cells that have accumulated at the tumor site. This was accomplished using a self-inactivating bicistronic lentivector that was engineered to produce human mAb-secreting CAR T cells [20, 23]. We used a mouse model of ccRCC to demonstrate proof-of-principle that anti-CAIX CAR T cells armed to deliver immuno-modulatory mAbs at the tumor site were superior to parental anti-CAIX CAR T cells in their ability to be resistant to exhaustion both *in vitro* and *in vivo*. As a result, a significant improvement in anti-tumor activity was seen *in vivo*. The ability of the secreted mAbs to recruit other immune cells to the tumor was also demonstrated.

Upregulation of PD-L1 on ccRCC cells is well documented [24] and believed to be involved in inducing T cell exhaustion through engagement of PD-1 on tumor recruited CD8+ T cells [25, 26]. Treatment of ccRCC with anti-CTLA4 and PD-L1/PD-1 checkpoint blockade inhibitors has shown some promising clinical results [27, 28]. Here, anti-CAIX CAR T cells that stably express and secrete anti-PD-L1 IgG1 or IgG4 mAbs were studied and compared to parental anti-CAIX CAR T cells and other cell controls. These anti-PD-L1 mAb-secreting CAR T cells maintained their capacity to undergo clonal expansion when in contact with CAIX+ RCC cells and to secrete high levels of IFNγ and IL-2. Remarkably, *in vitro* results showed that the secreted anti-PD-L1 IgG1 or IgG4 can interact with the PD-L1+ RCC cells, reverse upregulation of the exhaustion markers PD-1, TIM-3 and LAG-3 and restore tumor cell killing. Moreover, anti-CAIX CAR T cells secreting the IgG1 isotype of anti-PD-L1 were also able to induce ADCC *in vitro*.

In an orthotopic model of human ccRCC in NSG mice both anti-CAIX/anti-PD-L1 IgG1 and IgG4 CAR T cells were able to revert T cell exhaustion as evidenced by downregulation of exhaustion markers PD-1, TIM-3 and LAG-3 in the TILs. These anti-PD-L1-secreting anti-CAIX CAR T cells showed markedly enhanced anti-tumor effect with decreased tumor growth and weight when compared to parental anti-CAIX CAR T cells. In addition, both secreted anti-PD-L1 IgG1 or IgG4 are able to bind their target in the complex tumor milieu and induce PD-L1 downregulation. Moreover, the upregulation of granzyme B expression by anti-CAIX CAR T cells secreting anti-PD-L1 provides strong evidence for the restoration of killing activity of CAR T cells at the tumor site, and the decrease in Ki67 staining shows the reduction in the proliferation of tumor cells. While the mechanism of enhanced tumor cell killing is unknown, FcγR-bearing NSG myeloid cells can bind human IgG1 and IgG4 and may have some role in tumor cell clearance in the presence of anti-PD-L1 IgG1 or IgG4 isotypes [29]. The known expression of PD-L1 on activated CD8 T cells [30] together with the more potent killing of hIgG1- over hIgG4-treated target cells by murine PMNs may have contributed to the superiority of anti-PD-L1 IgG4-secreting anti-CAIX CAR T cells *in vivo*.

Another important proof-of-principle experiment involved the injection of human NK cells into the blood of mice one day before their euthanasia. Our previous experience with this orthotopic ccRCC model in NSG mice showed that human NK cells last only for a few days in the mouse blood [31], and for this reason the injection of these NK cells was not performed until the end of the experiment. The results demonstrated the specific recruitment of human NK cells into the tumor of mice treated with anti-PD-L1 IgG1-secreting anti-CAIX CAR T cells. The results also demonstrate that the locally secreted IgG1 is retained at the tumor site and is not rapidly diffused into the blood circulation. These results reflect a potential increase in the therapeutic efficacy of these CAR T cells when applied in humans, since the IgG1 binding to FcγRIIIa can induce NK-mediated ADCC. The local accumulation of NK cells can also facilitate the development of anti-tumor CD8+ cytotoxic T lymphocyte responses and increase the number of tumor-specific IFNγ-producing cells [32].

There is a growing census in the field of cancer immunotherapy that combination therapies will be required to achieve cancer “cures”. Indeed, the combination of anti-CTLA4 and anti-PD-1 mAb therapies have shown enhanced efficacy in clinical trials and appear to exert their effects on distinct populations of T cells [33–35]. In addition, in a HER2-transgenic mouse model of breast cancer, the group of mice treated with anti-HER2 CAR T cells and repeated injections of anti-PD-1 mAb after tumor implantation showed greater CD8+ CAR T cell function with less T cell exhaustion and enhanced tumor cell killing [36]. Our results demonstrate that armed second-generation anti-CAIX CAR T cells constitutively secreting anti-PD-L1 IgGs that are retained in the RCC milieu also show reduced T cell exhaustion and enhanced anti-tumor activity. It is possible that this single combination anti-cancer agent that delivers persistently high local concentrations of the anti-PD-L1 mAbs may provide a significant treatment advantage for solid tumors. The local expression of anti-PD-L1 mAb may also result in reduced systemic toxicity [37].

First-generation CAR T cells expressing a murine anti-CAIX scFv were previously developed and tested *in vivo* for the treatment of metastatic ccRCC [38–40]. The protocol that utilized multiple CAR T cell injections in association with IL-2 treatment resulted in toxicity comprised of limiting liver enzyme elevations caused by the recognition of CAIX expressed at low levels on bile duct epithelium. The potential mechanism(s) of this toxicity revealed the generation of both the generation of human antibodies as well as cell-mediated immunity against the murine scFv which may have contributed to the toxicity and promoted CAR T cells neutralization and clearance [40]. A later study was also performed in which pre-treatment of the patients with a low dose of the same humanized anti-CAIX mAb was used to attenuate the activity of CAR T cells against bile duct epithelial cells [38]. These results suggest that the use of a human anti-CAIX scFv and the newer generations of CAR vectors could greatly improve the efficiency of CAIX-targeted CAR T cells.

In conclusion, anti-CAIX CAR T cells secreting anti-PD-L1 IgG1 or IgG4 can diminish T cell exhaustion and improve CAR T cell treatment of ccRCC *in vivo*. We propose that modifications in the CAR structural designs such as the replacement of the co-stimulatory domain CD28 by 4–1BB may further enhance CAR T cell persistence *in vivo* and improve the efficiency of this treatment [41]. In addition, we foresee that a combination of mAbs that is directed to other molecules that are involved in immune escape, either expressed in tandem or as bi-specific mAbs, is among the next steps that should be explored to achieve even higher levels of anti-tumor immunity.

## MATERIALS AND METHODS

### Cell lines and culture

Human CAIX+/PD-L1- skrc52 and CAIX-/PD-L1+ skrc59 ccRCC cell lines were obtained from Dr. Gerd Ritter (Memorial Sloan-Kettering, NY). Cells were cultivated in RPMI 1640 Medium (Life Technologies) supplemented with 10% (v/v) heat-inactivated fetal bovine serum (FBS, Gibco), 100 IU/ml penicillin and 100 μg/ml streptomycin. CD8+ T cells were maintained in this complete RPMI medium containing 20 mM HEPES, and 50 IU/mL of IL-21 (Peprotech) was added in the medium every 2 days. 293T (CRL-11268, ATCC) and Lenti-X 293T (Clontech) cells were grown in DMEM Medium (Life Technologies) supplemented with 10% FBS, 100 IU/ml penicillin and 100 μg/ml streptomycin. Tumor cell lines were transduced with luciferase through lentiviral transduction and maintained at 37°C with 5% CO_2_. The skrc52 cells were selected for CAIX-/PD-L1- and CAIX+/PD-L1- cell populations by fluorescence activated cell sorting. Skrc59 cells were engineered to express high levels of human CAIX [42] and CAIX+/PD-L1+ were selected by sorting.

### Cloning of anti-PD-L1 scFv-Fc IgG1 and IgG4 into a bicistronic lentiviral vector encoding an anti-CAIX 2nd-generation CAR

The human anti-PD-L1 antibody (Ab) (clone 42) was previously selected using a 27 billion-member human single-chain variable fragment (scFv) antibody phage display library against a full-length PD-L1 in paramagnetic proteoliposomes [42, 43]. Clone 42 mAb was selected for this study due to its ability to efficiently block PD-L1/PD-1 binding (data not shown). The DNA sequences encoding the anti-PD-L1 scFv were codon optimized for mammalian expression, synthesized (Genewiz) with 5′ *NdeI* and 3′ *ClaI* restriction sites, and cloned in place of ZsGreen in the human-Fc IgG1 or stabilized IgG4 [44] lentiviral vector pHAGE-eIFa-leader-scFvG36(anti-CAIX)-C9TAG-linker-CD28-CD3z-IRES-ZsGreen [20]. For the negative controls, anti-PD-L1 scFv was replaced with an anti-severe acute respiratory syndrome (SARS) scFv (Clone 11A) in the IgG1-Fc lentiviral vector. The anti-CAIX scFv in the CAR construct containing the anti-SARS IgG1 was also replaced with the anti-B cell maturation antigen (BCMA). These procedures resulted in four constructs for lentivirus production: Anti-CAIX CAR able to express anti-PD-L1 IgG1 (anti-CAIX/anti-PD-L1 IgG1), anti-CAIX CAR able to express anti-PD-L1 IgG4 (anti-CAIX/anti-PD-L1 IgG4), anti-CAIX CAR able to express an irrelevant anti-SARS mAb (anti-CAIX/anti-SARS IgG1 – referred to as parental anti-CAIX CAR) and anti-BCMA CAR able to express an irrelevant anti-SARS mAb (anti-BCMA/anti-SARS IgG1).

### Production of lentivirus particles

Lentiviruses were produced by transient transfection of five plasmids into 293T cells using Polyethyleneimine (PEI) [45]. Briefly, each 80% confluent 293T cells in 15 cm plate (Nalge Nunc) was transfected with 30 μg of total five plasmids, being 5 μg of each structural plasmid pHDH-Hgpm2 (HIV gag-pol), pMD-tat; pRC/CMV-rev and Env VSV-G, and 10 μg of the CAR encoding plasmid (anti-CAIX/anti-PD-L1 IgG1, anti-CAIX/anti-PD-L1 IgG4, anti-CAIX/anti-SARS IgG1 or anti-BCMA/anti-SARS IgG1). The virus supernatant was concentrated using Lenti-X Concentrator (Clontech), following the manufacturer instructions, and kept frozen at −80°C.

### Selection, activation and lentivector transduction of CD8+ T cells

Blood collars collected from healthy volunteers were obtained from the blood bank of the Brigham and Woman's Hospital (Boston, MA) under a DFCI approved human protocol. Human peripheral blood mononuclear cells (PBMCs) were separated using Ficoll-Paque PLUS (GE Healthcare, NJ). The Dynabeads for CD8 Positive Isolation (Life Technologies) were used to isolate CD8 T cells from PBMCs, which were activated with Dynabeads Human T-Activator CD3/CD28 (Life Technologies) using a ratio of 1:1. Subsequently, the cells were transduced with the Lentiviruses at a multiplicity of infection of 20 and 10 μg/mL of Diethylaminoethyl. All the assays were performed in triplicate and using T cells from three different healthy donors. The ability of T cells to be transduced can be variable among different donors and, when this was the case, the number of cells added to the experiment was corrected by the % of transduction to avoid this bias.

### Flow cytometry

Transduction of cells was confirmed by analysis of cells stained with 10 μg/mL of human CAIX-human Fc produced in our lab or human BCMA-mouse-Fc (AB Bioscience) and then developed with 2 μg/mL APC-conjugated mouse anti-human IgG Ab (Southern Biotech) or goat-anti-mouse IgG Ab (Biolegend), respectively. CountBright^™^Absolute Counting Beads (Molecular Probes) was used for the proliferation and clonal expansion assays. All samples were analyzed with an LSR Fortessa or with a FACSCalibur (BD Bioscience) and data were analyzed using FlowJo software. To analyze the status of T cell exhaustion, the CAR T cells were cultured in the presence of IL-21 50 U/mL (Peprotech) and Dynabeads Human T Activator CD3/CD28 for 5 days. After this period the CAR T cells were co-cultured with skrc59 CAIX+/PD-L1+ cells for 9 days in order to stimulate exhaustion. 1 × 10^6^ CAR T cells from this assay or TIL collected from the *in vivo* assay were stained with FITC-conjugated anti-human PD-1, PE-conjugated anti-human TIM-3, PerCP/Cy5.5-conjugated anti-human LAG-3 antibodies (Biolegend) and Pacific Blue-conjugated anti-human CD45 and analyzed by flow cytometry. To verify the expression levels of CAIX and PD-L1 in the different RCC cell lineages, we used 10 μg/mL of the anti-human CAIX mAb (Clone G36) and 10 μg/mL of the biotinylated mouse anti-human PD-L1 (Biolegend). The primary antibodies were detected using 2 μg/mL APC-conjugated anti-human Ab (Southern Biotech) and 0.15 μg/mL of PE-conjugated avidin (Biolegend), respectively, and analyzed by FACS.

### Detection of IgG secreted by CAR T cells using ELISA

1 × 10^6^ CAR transduced CD8 T cells pre-activated with CD3/CD28 beads were maintained for two days in a 6 well plate containing 2 mL of complete RPMI 1640 medium (Life Technologies) and IL-21 50 U/mL. After 48 hours, 100 μL of the medium was removed to dose total IgG or anti-PD-L1 IgGs. Total level of IgG secreted into the medium of transduced cells was detected using Human IgG ELISA Quantitation Set (Bethyl Laboratories). Anti-PD-L1 Abs secreted by transduced CD8+ CAR T cells were purified with Protein A sepharose beads (GE Healthcare) and biotinylated using the EZ-Link Sulfo-NHS-LC-Biotin (Thermo Scientific). These antibodies were incubated with 5 μg/mL of recombinant PD-L1 human-Fc protein, which was pre-immobilized in MaxiSorp plates (Nunc) for 2 hours, RT. The biotinylated antibodies were detected by incubation with streptavidin-HRP for 1 h and developed with SureBlue^™^ TMB Peroxidase Substrate and TMB Stop Solution (KPL). The absorbance was read at λ = 450 nm.

### Clonal expansion of anti-CAIX CAR T cells

Skrc52 CAIX+/PD-L1- and skrc52 CAIX-/PD-L1- cells were irradiated with 3,000 rads and seeded at 2.5 × 10^5^ per well. 1 × 10^6^ CAR T cells were added to the culture medium containing 50 IU/ml human IL-21 every two days. T cells were split to maintain suitable density and re-stimulated with tumor cells weekly. T cell number was counted once a week for 3 weeks by FACS.

### Effect of anti-CAIX CAR T cells secreting anti-PD-L1 antibodies on RCC cells viability and antibody-dependent cellular cytotoxicity (ADCC)

2.5 × 10^3^ skrc59 CAIX+/PD-L1+ and skrc52 CAIX-/PD-L1- were plated in 96 wells plate overnight (ON). Four days after the CAR T cells transduction they were added to the RCC cells in the 25:1, 50:1 and 100:1 ratio Effector cells:Tumor cells (E:T) and incubated ON. CAR T cells were removed and the viability of tumor cells was assayed by MTT assay (Life Technologies). For the ADCC assay, RCC cells were incubated for 1 h, 37°C with 50 μL of the CAR T cells supernatant adjusted to 0.5 μg/mL of the respective Abs. The cells were then incubated with 12.5:1, 25:1 or 50:1 of NK cells:Tumor cells for 4 h, 37°C [46]. Lactate dehydrogenase (LDH) was measured in the supernatant by CytoTox 96^®^ Non-Radioactive Cytotoxicity Assay (Promega).

### ELISA assays to detect IL-2 and IFNγ released by functional CAR T cells

For analysis of cytokine secretion, 2.5 × 10^3^ RCC cells skrc59 CAIX+/PD-L1+ or skrc52 CAIX-/PD-L1-were seeded in 96 wells plates ON, followed by 5:1, 25:1 and 50:1 CAR T cells:Tumor cells addition and incubation ON. The supernatant was removed and analyzed for IL-2 and IFNγ secretion using the Human IFNγ or Human IL-2 ELISA Ready-SET-Go Kit (eBioscience).

### Establishment of the orthotopic renal cell carcinoma model and CAR T cell therapy

5 × 10^4^ skrc59 CAIX+/PD-L1+ cells were suspended in 10 μL of culture medium and diluted 1:1 in Matrigel^™^ (Life Technologies) and injected into the left subrenal capsule of 6–8 week-old male NOD/SCID/IL2Rγ−/− (NSG) mice (Jackson Laboratories). After a week, tumor implantation was confirmed by bioluminescence (BLI) image using a Xenogen IVIS imaging system (Life Technologies) and 1 × 10^7^ of each CAR T cell or untransduced T cells were injected intravenously in the tail vein (Day 0); *N* = 6 mice per group. The blood of 3 donors was used to generate CAR T cells from all groups and 2 mice of each group were injected with CAR T cells from a different donor. The tumor BLI was quantified after 7, 14, 23 and 30 days of CAR T cells injection. A second injection of 2.5 × 10^6^ CAR or untransduced T cells was made on Day 17. Mice from control groups presented signs of distress at 30 days post tumor engraftment and were sacrificed by standard CO_2_ inhalation. Tumors were harvested and weighed. The kidney tumors from all mice were divided in two equal parts and one of them was fragmented in small pieces and digested with collagenase 0.5 U/mL and DNAse 1.0 mg/mL for TIL extraction, which were analyzed for the expression of the exhaustion markers and the percentage of CAR T cells by flow cytometry. The other part was fixed in 10% buffered formaldehyde for immunohistochemistry (IHC). Two mice of each group were injected with 4.5 × 10^6^ NK cells 2 days before the euthanasia. NK cells present in the tumor were stained with APC-anti-CD56 Ab and analyzed by FACS.

### Immunohistochemistry staining and quantitation

Four-micrometer sections of formalin-fixed, paraffin-embedded tissues were prepared for conventional IHC and stained for 45 minutes with the rabbit anti-human granzyme B polyclonal Ab (Abcam, ab4059) 1:100, mouse anti-human PD-L1 mAb 10.4 μg/mL (Clone 405.9A11, (Boston, MA)) or rabbit anti-human Ki67 polyclonal Ab 1:2000 (Vector, VP-K451), followed by secondary HRP conjugated anti-rabbit or anti-mouse Ab. The anti-PD-L1 mAb used for IHC [21] recognizes a different domain than the anti-PD-L1 mAb secreted by the lentivirus. The slides were developed using 3,3′-diaminobenzidine (DAB) and counterstained with hematoxylin. The quantification of the IHC images was performed using the IHC Profiler Plugin of ImageJ Software [47].

### Statistical analysis

The statistical significance of the data was evaluated using ANOVA and Tukey post test using the IBM SPSS Statistics software version 20. *P* < 0.05 was considered significant.

## SUPPLEMENTARY MATERIALS FIGURES


