# Supplementary material for: Chimeric antigen receptor T cells secreting anti-PD-L1 antibodies more effectively regress renal cell carcinoma in a humanized mouse model
Source: Oncotarget. 2016 Apr 29;7(23):34341–55. doi: 10.18632/oncotarget.9114 (PMC5085160; doi:10.18632/oncotarget.9114)
Supplement: Supplementary file 1 [file oncotarget-07-34341-s001.pdf]

Chimeric antigen receptor T cells secreting anti-PD-L1 antibodies more effectively regress renal cell carcinoma in a humanized mouse model

Supplementary Materials

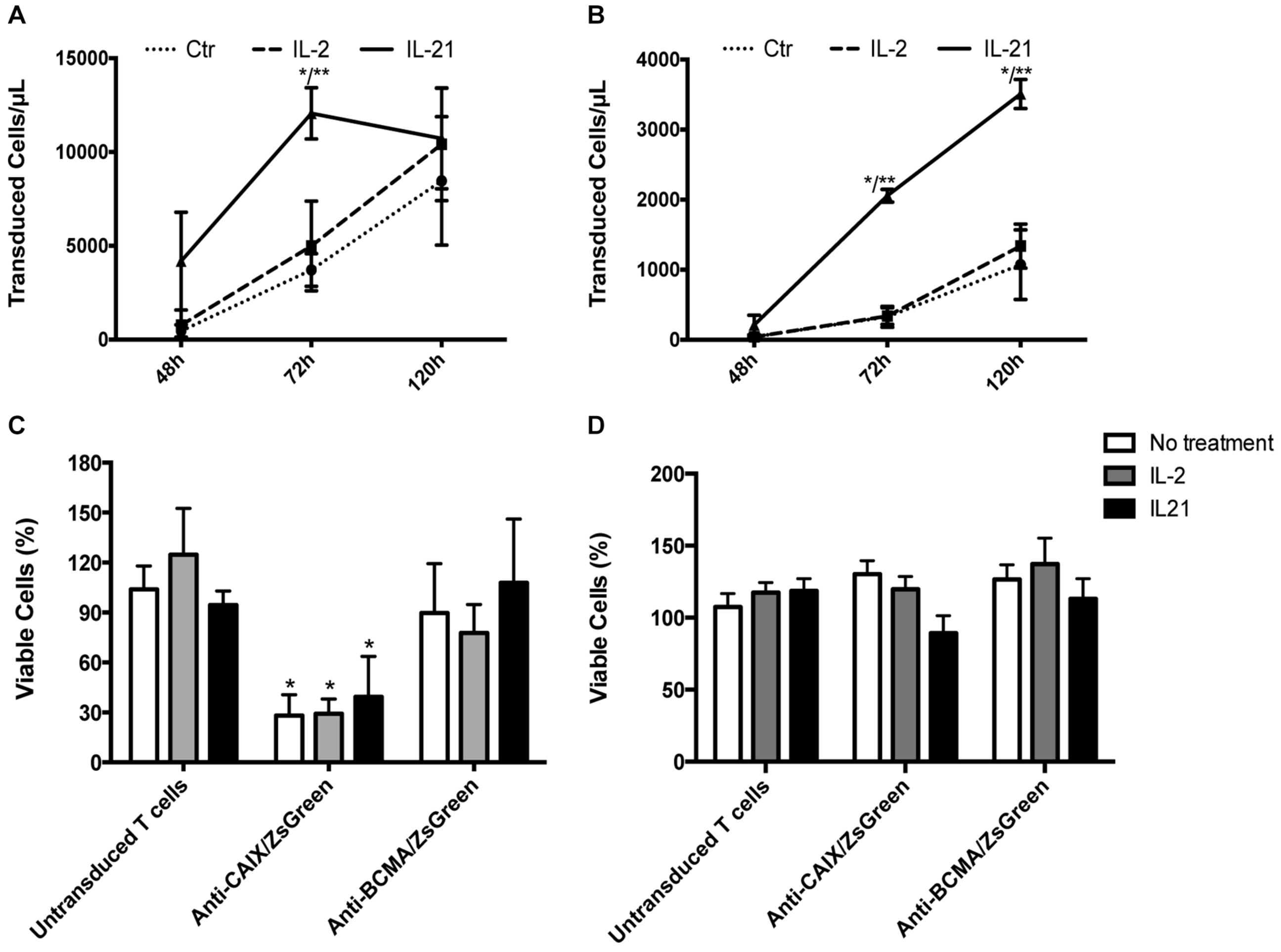

Supplementary Figure S1: Evaluation of IL-2 versus IL-21 to CD8+ CAR T cells proliferation. (**A** and **B**) Proliferation of CAR T transduced cells in the presence of IL-2 or IL-21 evaluated 48 h, 72 h and 120 h after transduction with lentiviruses. (A) Anti-CAIX CAR T cells or (B) irrelevant anti-BCMA CAR T cells (both with ZsGreen in the second cassette). The CD8+ T cells were selected using Dynabeads CD8 Positive Isolation Kit (Life Technologies) and activated with Dynabeads Human T Activator CD3/CD28 (Life Technologies) in the presence of IL2 or IL-21 50 U/mL (Peprotech). The CAR T cells transduction was evaluated by ZsGreen expression using FACS. The data represents the average ± SD of two donors. *$p < 0.05$ comparing IL-21 with non treated control (Ctr); **$p < 0.05$ comparing IL21 with IL-2. (**C** and **D**) Viability of RCC cells treated with CD8+ CAR T cells cultivated in the presence of IL-2 or IL-21. The viability was evaluated by MTT after an overnight incubation of anti-BCMA, anti-CAIX CAR T cells or untransduced T cells with (C) skrc59 CAIX+/PD-L1+ and (D) skrc52 CAIX-/PD-L1- RCC cells. The CAR T cells were previously cultured in the presence of IL2 or IL-21 50 U/mL for 120 hours. These results represent the average ± SD of two donors in triplicate. *$P < 0.05$ comparing anti-CAIX CAR with anti-BCMA CAR or untransduced T cells.

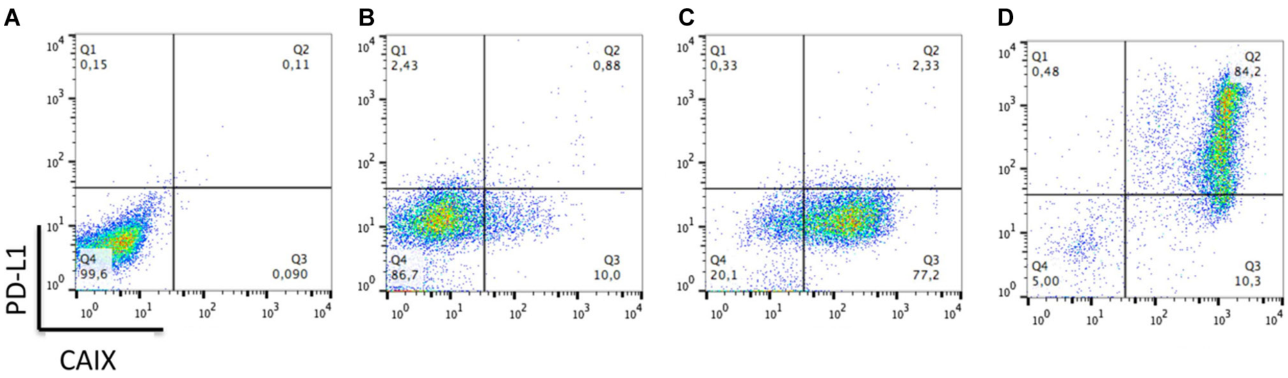

Supplementary Figure S2: Expression of PD-L1 and CAIX in the RCC lines. (**A**) Negative control (skrc59 CAIX+/PD-L1+ stained with secondary antibodies only), (**B**) skrc52 CAIX-/PD-L1-, (**C**) skrc52 CAIX+/PD-L1-, (**D**) skrc59 CAIX+/PD-L1+. The cells were stained with anti-human CAIX antibody followed by the secondary APC-anti-human Fc IgG and biotinylated anti-human PD-L1 antibody labeled with PE-Avidin. The flow cytometry analysis was then performed.

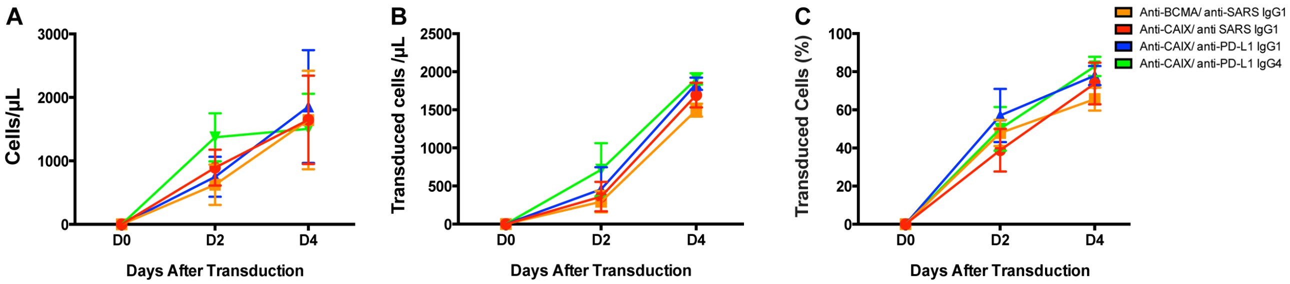

Supplementary Figure S3: CAR T cells characterization. (**A**) Proliferation of total CD8+ T cells two or four days after transduction with lentivector encoding anti-CAIX CAR/anti-PD-L1 IgG1, anti-CAIX CAR/anti-PD-L1 IgG4, anti-CAIX CAR/anti-SARS IgG1 or anti-BCMA CAR/anti-SARS IgG1). The CD8+ T cells were selected using Dynabeads CD8 Positive Isolation Kit (Life Technologies) and activated with Dynabeads Human T Activator CD3/CD28 (Life Technologies) in the presence of IL-21 50 U/mL. IL-21 was added to the medium every 2 days. The proliferation was evaluated by flow cytometry with Counting Beads (Molecular Probes). (**B**) Concentration of CAR-transduced T cells two and fours days after transduction. The CAR T cells were incubated with soluble human CAIX-Fc or BCMA-Fc, followed by incubation with an APC conjugated anti-human Fc IgG and analyzed by flow cytometry. (**C**) Percentage of CAR T cells 2 and 4 days after transduction. The results represent the average ± SD of three donors in duplicate.

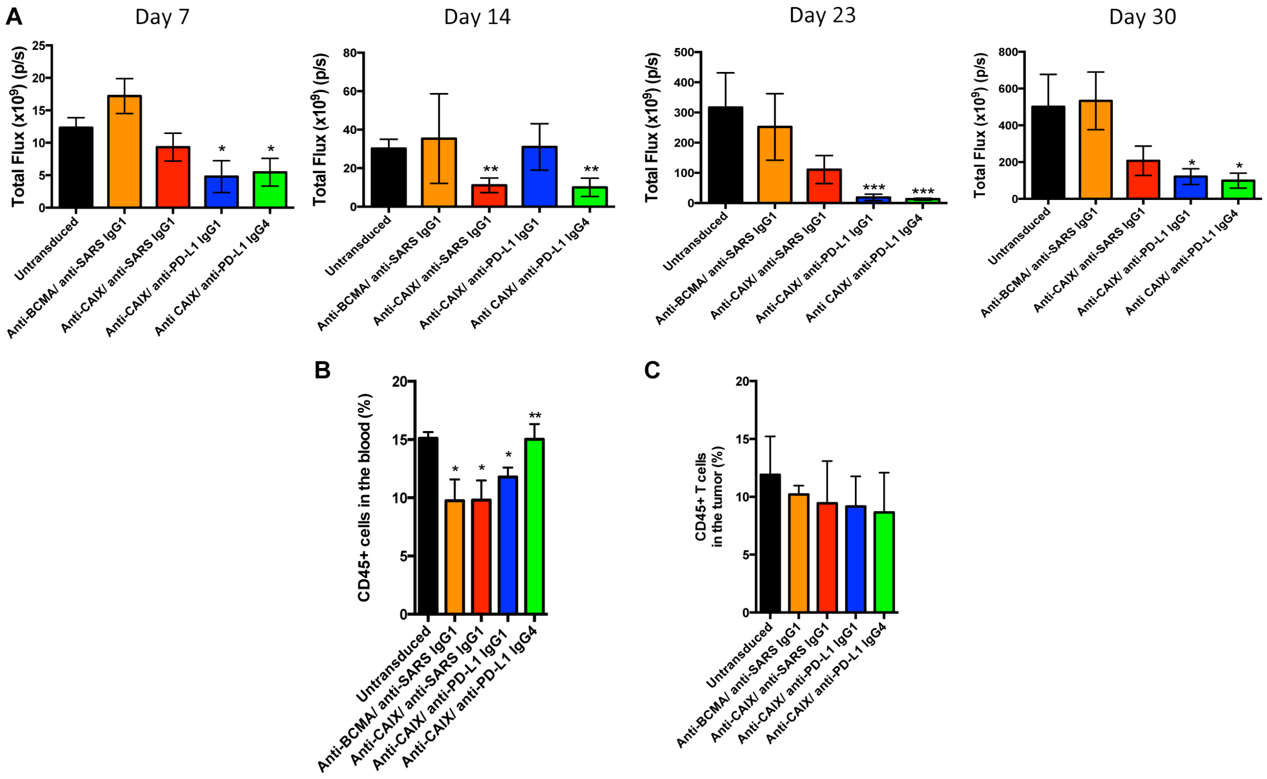

Supplementary Figure S4: CAR T cells effect in an orthotopic model of human RCC. (**A**) Comparison of tumor size detected quantified by bioluminescence versus days among the CAR T cells groups. NSG Mice were injected under the renal capsule with 5.0×10^4 skrc59 CAIX+/PD-L1+/luciferase+ RCC cells. The mice were injected with 1.0×10^7 CAR T or untransduced T cells by i.v. on Day 7 post tumor implantation, then again on Day 17 with a second treatment of 2.5×10^6 CAR T cells. The CAR T cells were previously transduced with the following lentiviral vectors encoding: anti-BCMA CAR/anti-SARS IgG1, anti-CAIX CAR/anti-SARS IgG1, anti-CAIX CAR/anti-PD-L1 IgG1 and anti-CAIX CAR/anti-PD-L1 IgG4 ($N = 6$ mice per group). The tumor bioluminescence was quantified after 5 minutes of luciferin IP injection using IVIS. *$P < 0.05$ compared to anti-BCMA CAR T cells. **$P < 0.05$ compared to untransduced T cells, ***$P < 0.05$ compared to anti-CAIX CAR/anti-SARS IgG1. (**B**) Percentage of human T cells in the mice blood after 8 days of treatment. *$P < 0.05$ compared to untransduced T cells. **$P < 0.05$ compared to all anti-CAIX CARs. The red blood cells were lysed with ACK Lysing Buffer (Lonza) and the remaining cells were stained with Pacific Blue conjugated anti-human CD45 and analyzed by flow cytometry. (**C**) Total tumor-infiltrating lymphocytes (TIL) after 30 days of treatment with the CAR T cells. The tumors and kidney from all mice were divided in two parts and one of them was fragmented in small pieces and digested with collagenase and DNAse to extract TIL. The cells were stained with Pacific Blue conjugated anti-human CD45 and analyzed by flow cytometry.
